# *ALKBH5* Gene Polymorphisms and Hepatoblastoma Susceptibility in Chinese Children

**DOI:** 10.1155/2021/6658480

**Published:** 2021-03-19

**Authors:** Hui Ren, Zhen-Jian Zhuo, Fei Duan, Yong Li, Zhonghua Yang, Jiao Zhang, Jiwen Cheng, Suhong Li, Li Li, Jianlei Geng, Zhiguang Zhang, Jing He, Huizhong Niu

**Affiliations:** ^1^Department of Pediatric General Surgery, Hebei Children's Hospital of Hebei Medical University, Shijiazhuang 050031, Hebei, China; ^2^Department of Pediatric Surgery, Guangzhou Institute of Pediatrics, Guangdong Provincial Key Laboratory of Research in Structural Birth Defect Disease, Guangzhou Women and Children's Medical Center, Guangzhou Medical University, Guangzhou 510623, Guangdong, China; ^3^Department of Pediatric Surgery, Hunan Children's Hospital, Changsha 410004, Hunan, China; ^4^Department of Pediatric Surgery, Shengjing Hospital of China Medical University, Shenyang 110004, Liaoning, China; ^5^Department of Pediatric Surgery, The First Affiliated Hospital of Zhengzhou University, Zhengzhou 450052, Henan, China; ^6^Department of Pediatric Surgery, The Second Affiliated Hospital of Xi'an Jiaotong University, Xi'an 710004, Shaanxi, China; ^7^Department of Pathology, Children Hospital and Women Health Center of Shanxi, Taiyuan 030013, Shaanxi, China; ^8^Kunming Key Laboratory of Children Infection and Immunity, Yunnan Key Laboratory of Children's Major Disease Research, Yunnan Institute of Pediatrics Research, Yunnan Medical Center for Pediatric Diseases, Kunming Children's Hospital, Kunming 650228, Yunnan, China

## Abstract

Incidence of hepatoblastoma has been increasing, but the causes of this disease remain unclear. Some studies have suggested that abnormal expressions of *ALKBH5* gene are associated with multiple cancers. This study aims to test the hypothesis that hepatoblastoma risk may be modulated by genetic polymorphisms in *ALKBH5* gene based on genotyped data from samples of 328 cases and 1476 controls enrolled from eight hospitals in China. We used TaqMan assay to genotype *ALKBH5* gene single nucleotide polymorphisms (SNPs) rs1378602G > A and rs8400G > A. We calculated the odds ratios (ORs) and *P* values using logistic regression models to estimate the association between hepatoblastoma risk and *ALKBH5* gene SNPs. We found the rs1378602G > A and rs8400G > A could not impact hepatoblastoma risk in single or combined analysis. Stratified analysis revealed that subjects with the rs8400 AA genotype are prone to getting hepatoblastoma in the clinical stage III + IV subgroup (adjusted OR = 1.93, 95% CI = 1.20–3.10, *P*=0.007), when compared to those with GG/GA genotype. False-positive report probability validated the reliability of the significant results. Preliminary functional annotations revealed that rs8400 A is correlated with increased expression of *ALKBH5* gene in the expression quantitative trait locus (eQTL) analysis. In all, our investigation presents evidence of a weak impact of *ALKBH5* gene polymorphisms on hepatoblastoma risk, using the largest hepatoblastoma sample size. These findings shed some light on the genetic basis of hepatoblastoma, implicating the role of *ALKBH5* gene polymorphisms in the etiology of hepatoblastoma.

## 1. Introduction

Hepatoblastomas are extremely rare, accounting for only 1% of all malignancies in the pediatric age group [1]. Although its incidence has increased markedly over the decades, it only accounts for about 1.5 cases per million children per year [2]. However, hepatoblastoma ranks the most frequent liver malignancy in childhood, with up to about 80% of all children's hepatic tumors [3, 4]. Dramatic improvements in clinical treatment have led to an increase in prognosis, with a five-year survival rate above 80% [5]. For clinically advanced hepatoblastomas, there are limited treatment options by now. What is worse, some patient survivors may suffer severe and lifelong side effects after immunosuppression and intensive chemotherapy [6].

The origin of hepatoblastoma is largely unknown [7]. Unlike adult hepatocellular carcinoma (HCC), hepatoblastoma develops in the absence of hepatitis B virus, chronic hepatitis, and cirrhosis [8]. Hepatoblastoma generally presents with a large abdominal mass, activating mutations of the *catenin beta 1* (*CTNNB1*) gene, and an elevated *α*-fetoprotein protein (AFP) level [9–18]. However, the genetic determinants of hepatoblastoma are still unclear. To date, only a few case-control studies have investigated the role of single nucleotide polymorphisms (SNPs) on hepatoblastoma risk, with sample sizes of less than 100 [19, 20]. To solve this dilemma, our group has been working along with other centers to recruit as many hepatoblastoma cases as possible to shed light on the genetic etiology of hepatoblastoma. We have found that SNPs in *LINC00673*, *NRAS*, *KRAS*, and *TP53* genes may modify the risk of hepatoblastoma in the Chinese population [21–23]. The rarity of the disease has impaired the adequate investigation of their etiology. Therefore, new insights into the biology of hepatoblastoma are needed.

N6-Methyladenosine (m^6^A) is the most abundant mRNA posttranscriptional modification [24, 25]. m^6^A modification is reversible and relies on the m^6^A methyltransferase (“writers”: METTL3, METTL14, and WTAP), m^6^A demethylases (“erasers”: FTO and ALKBH5), and m^6^A-binding proteins (“readers”: IGF2BP1 and YTHDF1) [26–29]. Emerging evidence indicates that m^6^A modification is involved in a plethora of physiological and pathological processes, including carcinogenesis [30]. Alkylation repair homolog protein 5 (ALKBH5) functions as a key demethylase in mediating methylation reversal. The aberrant function of ALKBH5 contributes to several types of cancers via specific mechanisms [31]. While *ALKBH5* functions as a probably carcinogenic or cancer suppressor gene in humans and several epidemiological studies reported associations between *ALKBH5* gene polymorphisms and cancer, no previous studies were reported on the association between *ALKBH5* gene polymorphisms and hepatoblastoma risk. To discover more variants predicting the risk of hepatoblastoma, here we conducted a multicenter case-control study among children of Chinese ancestry.

## 2. Materials and Methods

### 2.1. Study Subjects

Details of the selection of subjects and collection of specimens have been published elsewhere [22, 23]. This is a collaborative study with hepatoblastoma cases and controls enrolled from eight hospitals in China. Eligible cases were children newly diagnosed with a histologically confirmed hepatoblastoma and residing in the corresponding areas of the hospital at diagnosis. Eligibility criteria for controls were Chinese and no underlying medical disorder, including cancer. They were residing in the same areas as the cases during the same period. The specimens were annotated with clinical and genomic information containing age, sex, and clinical stages at diagnosis. In total, 328 cases of hepatoblastoma and 1476 hospital-based controls were enrolled in the study. All participating studies were approved by the relevant ethics committees and informed consent was obtained from each study participant.

### 2.2. Genotyping

Two SNPs, rs1378602G >  A nor rs8400G > A, were screened out for analysis with the criteria described in our previous study [32]. Blood samples of patients were taken at the time of diagnosis of hepatoblastoma and then proceeded to genomic DNA extraction by QIAamp DNA Blood Mini Kit (QIAGEN Inc., Valencia, CA) shortly thereafter. The quality and quantity of the DNA samples were assessed using NanoDrop 2000 (ThermoFisher Scientific, Waltham, MA). We genotyped the samples using the TaqMan assay on an ABI 7900 (Applied Biosystems, Foster City, CA, USA) following the procedure recommended by the vendor. Detailed procedures of genotyping and data quality control have been described in a previous study [33, 34]. Laboratory personnel was blinded to the sample categories including case-control status and quality control.

### 2.3. Statistical Analysis

Chi-square test or *t*-test, as appropriate, was analyzed to compare the characteristics differences of the two groups. We adopted a goodness-of-fit *χ*^2^ test to assess whether individual SNPs in controls conform to the Hardy–Weinberg equilibrium (HWE). A logistic regression model was used to examine the associations of interested SNPs and hepatoblastoma risk adjusting for covariates. The strength of association was determined by odds ratios (ORs) and 95% confidence intervals (CIs). False-positive report probability (FPRP) analysis was conducted to assess noteworthy associations. Expression quantitative trait loci (eQTL) analysis using GTEx portal website (http://www.gtexportal.org/home/) was conducted to determine the correlation between the SNPs and its locating gene expression. All tests were two-sided; statistical significance used an alpha of 0.05. SAS software version 9.1 (SAS, US) was used for statistical analysis.

## 3. Results

### 3.1. Characteristics of the Participants

In Table S1, we described the distribution of age, sex, and clinical stages among 328 cases and 1476 controls. Cases have a similar distribution in terms of age and gender to that of controls, with *P*=0.275 and *P*=0.964, respectively. For clinical stages, 99 (30.18%), 74 (22.56%), 65 (19.82%), 28 (8.54%), and 62 (18.90%) were diagnosed with stages I, II, III, IV, and NA.

### 3.2. Association between the *ALKBH5* SNPs and Hepatoblastoma Risk

We successfully genotyped the *ALKBH5* gene SNPs in samples of 324 cases and 1476 controls among the included 328 cases and 1476 controls. The detailed information regarding the relationship between *ALKBH5* gene SNPs with hepatoblastoma risk was shown in Table 1. We did not observe any violation of SNPs to the Hardy–Weinberg equilibrium in control populations (all *P* values >0.05). Neither rs1378602G > A nor rs8400G > A showed suggestive evidence of association with hepatoblastoma risk. We assumed rs1378602 AA and rs8400GA/GG genotypes as risk genotypes to further evaluate their combined effect on the risk of hepatoblastoma. Carriers with 1, 2, or 1-2 risk genotypes had similar hepatoblastoma incidence when compared to those without the risk genotype.

### 3.3. Stratification Analysis

We stratified the analysis by age, sex, and clinical stage of the subjects (Table 2). Under any subgroup analyzed, rs1378602GA/AA and 1-2 risk genotypes could not impact hepatoblastoma risk, when compared to their reference genotypes. Regarding SNP rs8400, AA genotype was found to be significantly associated with hepatoblastoma risk in children with clinical stage III + IV (adjusted OR = 1.93, 95% CI = 1.20–3.10, *P*=0.007).

### 3.4. False-Positive Report Probability (FPRP) Analysis

FPRP analysis was carried out to interrogate the significant findings (Table S2). The threshold for FPRP was preset as 0.2. At the prior probability level of 0.25, findings for stage III + IV in rs8400 AA versus GG/GA remained noteworthy.

### 3.5. Expression Quantitative Trait Loci (eQTL) Analyses

We further explored whether rs8400G > A could influence the mRNA level of its locating gene using released data from GTEx. We found that the rs8400 A genotype was significantly associated with higher ALKBH5 mRNA level when compared to the rs8400 G genotype (Figure S1).

## 4. Discussion

The extreme rarity of hepatoblastoma has limited the epidemiological investigation on the risk of hepatoblastoma. Here, we carried out by far the largest study to determine whether *ALKBH5* gene SNPs could predispose to hepatoblastoma risk in Chinese children. We identified SNP rs8400 with suggestive evidence of a weak association with hepatoblastoma risk. Our findings support the concept that genetic variation of *ALKBH5* gene may be implicated in the pathogenesis of hepatoblastoma.

Two main m^6^A demethylases have been identified by far: fat mass and obesity-associated protein (FTO) and ALKHB5. ALKHB5 belongs to the AlkB family [35]. In 2013, ALKBH5 was firstly found to have the ability to remove m6A methylation [29]. Knockdown of ALKBH5 significantly enhanced RNA m^6^A levels and promoted the RNAs exportation from the nucleus to the cytoplasm [29]. In addition, ALKBH5 also participates in mRNA processing and RNA metabolism [29]. ALKBH5 functions differently in specific cancers, as an oncogenic or tumor suppressor by mediating specific mechanisms. A lower ALKBH5 level was detected in hepatocellular carcinoma (HCC), indicating its prognostic factor of worse survival in HCC patients. Mechanistically, ALKBH5 acts as a tumor suppressor by inhibiting LY6/PLAUR Domain Containing 1 (LYPD1) via a m6A-dependent manner in HCC cells [36]. ALKBH5 also acts as a tumor suppressor in non-small cell lung cancer (NSCLC) [37]. Zhang et al. [38] found that overexpression of ALKBH5 could upregulate *FOXM1* gene expression and then promote self-renewal and proliferation of glioblastoma stem-like cells (GSCs). Shen et al. [39] found that increased ALKBH5 expression is correlated with poor prognosis in acute myeloid leukemia (AML) patients. Mechanistically, ALKBH5 exerts a protumorigenic role in AML by posttranscriptional regulation of its critical target TACC3. Panneerdoss et al. [40] demonstrated that ALKBH5 exerts its tumor-promoting effects by perturbing the normal expression m^6^A level of relevant pathway genes.

Although the significance of m^6^A modification genes in cancer is highly appreciated, the study of m^6^A modification gene SNPs is a nascent field as yet. *FTO* gene SNPs have been intensively reported to contribute to the risk of multiple human malignancies, including obesity and cancer [41–43]. However, the information regarding *ALKBH5* gene SNPs is still limited. Only recently has it begun to be realized that *ALKBH5* gene SNPs contribute to the genetic predisposition of cancer. In 2019, Meng et al. published the first case-control study on m^6^A modification gene SNPs and colorectal cancer risk. They comprehensively genotyped 240 SNPs in 20 m^6^A modification-related genes in the Chinese population. Only one SNP, rs118049207, located in the *SND1* gene, was significantly correlated with colorectal cancer risk. Mechanically, rs118049207 could alter the expression level of the *SND1* gene and then lead to the aberrant m^6^A level. However, all analyzed *ALKBH5* SNPs (rs2124370, rs8400, rs9899249, rs9913266, and rs2925137) could not predispose to colorectal cancer [44]. We have previously genotyped two SNPs (rs1378602 and rs8400) in the *ALKBH5* gene to identify if they were associated with Wilms tumor susceptibility in a large multicenter case-control study. Each SNP failed to contribute to Wilms tumor susceptibility. Stratification analysis did reveal some significant relationships between these SNPs and Wilms tumor risk in certain subgroups, indicating a weak influence of *ALKBH5* gene SNPs on susceptibility to Wilms tumor [32].

Given the important role of *ALKBH5* gene in cancer as well as the lack of research on this gene in hepatoblastoma, we performed the current study to investigate the association between *ALKBH5* gene SNPs and the risk of hepatoblastoma. The current analysis revealed that rs1378602G > A and rs8400G > A polymorphisms were not significantly associated with hepatoblastoma risk. The combined risk genotype analysis still did not reveal a significant relationship, indicating their relatively weak impact on hepatoblastoma risk. Regarding SNP rs8400, AA genotype was found to be significantly associated with hepatoblastoma risk in children in clinical stage III + IV. FPRP analysis further validated the strength of the significant findings. We further attempted to interpret the possible mechanism of ALKBH5 gene rs8400G > A-mediated hepatoblastoma risk. eQTL evidence suggested that the A allele in rs8400 is significantly associated with increased ALKBH5 level. Further functional experiments are needed to elucidate this mechanism. The negative relationship detected in the current study may be attributed to the limited statistical power of the moderate sample size, modification of other potential pertinent factors, and the weak effects of these two SNPs. Therefore, continuing studies with a much larger sample size as well as the inclusion of other interacting factors is warranted.

A major strength of the current study is the large sample size, which is generally representative of the Chinese population since the participants were recruited from eight major hospitals in China. Some potential limitations still exist. First, the number of patients in the subgroup analysis was relatively low, which perhaps precludes a robust statistical conclusion concerning these subgroups at present. Furthermore, we could not evaluate the effect of environmental effects on hepatoblastoma risk. If these factors greatly impact hepatoblastoma risk, the effectiveness of the variants here might have been overestimated. Third, although we included two SNPs in the *ALKBH5* gene, we lacked the power to assess more SNPs. In addition, the conclusion here fits the Chinese population; caution should be taken when applied to other ethnicities.

## 5. Conclusion

In summary, our study provides some clues of a potential modulating effect of *ALKBH5* gene SNPs on hepatoblastoma risk. A better understanding of the role of *ALKBH5* gene SNPs in the pathogenesis of hepatoblastoma might lead to new methods for the prevention and treatment of this disease.

## Figures and Tables

**Table 1:** Association between *ALKBH5* gene polymorphisms and hepatoblastoma risk.

Genotype	Cases (*N* = 324)	Controls (*N* = 1476)	*P* ^a^	Crude OR (95% CI)	*P*	Adjusted OR (95% CI)^b^	*P* ^b^
rs1378602G>A (HWE = 0.373)							
GG	277 (85.49)	1210 (81.98)		1.00		1.00	
GA	43 (13.27)	256 (17.34)		0.73 (0.52–1.04)	0.082	0.73 (0.52–1.03)	0.076
AA	4 (1.23)	10 (0.68)		1.75 (0.54–5.61)	0.349	1.76 (0.55–5.67)	0.341
Additive			0.234	0.83 (0.61–1.13)	0.235	0.82 (0.60–1.13)	0.225
Dominant	47 (14.51)	266 (18.02)	0.131	0.77 (0.55–1.08)	0.132	0.77 (0.55–1.08)	0.124
Recessive	320 (98.77)	1466 (99.32)	0.301	1.83 (0.57–5.88)	0.309	1.85 (0.58–5.95)	0.301

rs8400G>A (HWE = 0.063)							
GG	93 (28.70)	464 (31.44)		1.00		1.00	
GA	164 (50.62)	758 (51.36)		1.08 (0.82–1.43)	0.592	1.08 (0.82–1.43)	0.600
AA	67 (20.68)	254 (17.21)		1.32 (0.93–1.87)	0.123	1.31 (0.92–1.86)	0.125
Additive			0.141	1.14 (0.96–1.36)	0.141	1.14 (0.96–1.36)	0.143
Dominant	231 (71.30)	1012 (68.56)	0.335	1.14 (0.87–1.48)	0.336	1.14 (0.87–1.48)	0.342
Recessive	257 (79.32)	1222 (82.79)	0.140	1.25 (0.93–1.70)	0.140	1.25 (0.93–1.69)	0.141

Combined effect of risk genotypes^c^							
0	93 (28.70)	464 (31.44)		1.00		1.00	
1	227 (70.06)	1002 (67.89)		1.13 (0.87–1.47)	0.365	1.13 (0.87–1.47)	0.372
2	4 (1.23)	10 (0.68)	0.260	2.00 (0.61–6.50)	0.251	2.01 (0.62–6.57)	0.246
0	93 (28.70)	464 (31.44)		1.00		1.00	
1-2	231 (71.30)	1012 (68.56)	0.335	1.14 (0.87–1.48)	0.336	1.14 (0.87–1.48)	0.342

OR, odds ratio; CI, confidence interval; HWE, Hardy–Weinberg equilibrium. ^a^*χ*^2^ test for genotype distributions between hepatoblastoma patients and cancer-free controls. ^b^Adjusted for age and sex. ^c^Risk genotypes were carriers with rs1378602 AA and rs8400GA/GG genotypes.

**Table 2:** Stratification analysis for the association between *ALKBH5* genotypes and hepatoblastoma susceptibility.

Variables	rs1378602 (case/control)		AOR (95% CI)^a^	*P* ^a^	rs8400 (case/control)		AOR (95% CI)^a^	*P* ^a^	Combine genotypes (case/control)		AOR (95% CI)^a^	*P* ^a^
	GG	GA/AA			GG/GA	AA			0	1-2		
Age, month												
<17	151/532	24/129	0.65 (0.41–1.05)	0.077	141/550	34/111	1.21 (0.79–1.86)	0.381	54/206	121/455	1.02 (0.71–1.47)	0.908
≥17	126/678	23/137	0.90 (0.55–1.45)	0.651	116/672	33/143	1.34 (0.88–2.06)	0.177	39/258	110/557	1.31 (0.88–1.94)	0.181

Sex												
Female	115/485	17/120	0.60 (0.35–1.03)	0.065	108/495	24/110	1.00 (0.61–1.63)	0.998	41/184	91/421	0.97 (0.64–1.45)	0.872
Male	162/725	30/146	0.91 (0.59–1.39)	0.648	149/727	43/144	1.46 (0.99–2.14)	0.054	52/280	140/591	1.28 (0.91–1.82)	0.161

Clinical stages												
I + II	149/1210	24/266	0.72 (0.46–1.14)	0.159	147/1222	26/254	0.85 (0.55–1.32)	0.462	50/464	123/1012	1.12 (0.79–1.59)	0.512
III + IV	78/1210	13/266	0.77 (0.42–1.40)	0.387	65/1222	26/254	**1.93 (1.20–3.10)**	**0.007**	25/464	66/1012	1.21 (0.76–1.95)	0.425

AOR, adjusted odds ratio; CI, confidence interval. ^a^Adjusted for age and sex, omitting the corresponding stratify factor.

## Data Availability

All the data are available upon request.

## Abbreviations

HCC: Hepatocellular carcinoma
CTNNB1: Catenin beta 1
AFP: *α*-Fetoprotein protein
SNP: Single nucleotide polymorphism
m^6^A: N6-Methyladenosine
ALKBH5: Alkylation repair homolog protein 5
HWE: Hardy–Weinberg equilibrium
OR: Odds ratio
CI: Confidence interval
FTO: Obesity-associated protein
LYPD1: LY6/PLAUR Domain Containing 1
NSCLC: Non-small cell lung cancer
GSCs: Glioblastoma stem-like cells
AML: Acute myeloid leukemia
FPRP: False-positive report probability analysis
eQTL,: Expression quantitative trait locus analysis.

## Supplementary Materials

Supplementary Materials Table S1: frequency distribution of selected variables in hepatoblastoma patients and cancer-free controls from eight hospitals. Table S2: false-positive report probability analysis for significant finding in the stratify analysis. Figure S1: functional implication of rs8400 polymorphism based on the public database GTEx Portal. The genotype of rs8400 and expression of *ALKBH5* gene in brain cortex.
