# Supplementary material for: ALKBH5 Gene Polymorphisms and Hepatoblastoma Susceptibility in Chinese Children
Source: J Oncol. 2021 Mar 19;2021:6658480. doi: 10.1155/2021/6658480 (PMC7997766; doi:10.1155/2021/6658480)
Supplement: Supplementary Materials — Table S1: frequency distribution of selected variables in hepatoblastoma patients and cancer-free controls from eight hospitals. Table S2: false-positive report probability analysis for significant finding in the stratify analysis. Figure S1: functional implication of rs8400 polymorphism based on the public database GTEx Portal. The genotype of rs8400 and expression of ALKBH5 gene in brain cortex. [file 6658480.f1.doc]

**Supplementary Information**

| **Table S1**. Frequency distribution of selected variables in hepatoblastoma patients and cancer-free controls from eight hospitals | | | | | |
| --- | --- | --- | --- | --- | --- |
| Variables | Cases (n=328) | | Controls (n=1476) | | *P* ^a^ |
|  | No. | % | No. | % |  |
| Age range, month | 0.03-149.97 | | 0.004-156.00 | |  |
| Mean ± SD | 23.42 ± 25.51 | | 25.06 ± 19.32 | | 0.275 ^b^ |
| <17 | 178 | 54.27 | 661 | 44.78 |  |
| ≥17 | 150 | 45.73 | 815 | 55.22 |  |
| Sex |  |  |  |  | 0.964 |
| Female | 134 | 40.85 | 605 | 40.99 |  |
| Male | 194 | 59.15 | 871 | 59.01 |  |
| Clinical stages |  |  |  |  |  |
| I | 99 | 30.18 | / | / |  |
| II | 74 | 22.56 | / | / |  |
| III | 65 | 19.82 | / | / |  |
| IV | 28 | 8.54 | / | / |  |
| NA | 62 | 18.90 | / | / |  |
| SD, standard deviation, NA, not available.  ^a^ Two-sided χ^2^ test for distributions between hepatoblastoma cases and cancer-free controls.  ^b^ T-test for age distribution between hepatoblastoma patients and cancer-free controls. | | | | | |

| **Table S2**. False-positive report probability analysis for significant finding in the stratify analysis | | | | | | | | |
| --- | --- | --- | --- | --- | --- | --- | --- | --- |
| Variable | OR (95% CI) | *P* ^a^ | Statistical  power ^b^ | Prior probability | | | | |
|  |  |  |  | 0.25 | 0.1 | 0.01 | 0.001 | 0.0001 |
| rs8400 AA vs. GG/GA | | | | | | | | |
| Stage III+IV | 1.92 (1.20-3.09) | 0.007 | 0.159 | 0.114 | 0.278 | 0.809 | 0.977 | 0.998 |
| OR, odds ratio; CI, confidence interval.  ^a^ Chi-square test was used to calculate the genotype frequency distributions.  ^b^ Statistical power was calculated using the number of observations in each subgroup and the corresponding ORs and *P* values in this table. | | | | | | | | |

**Figure S1**


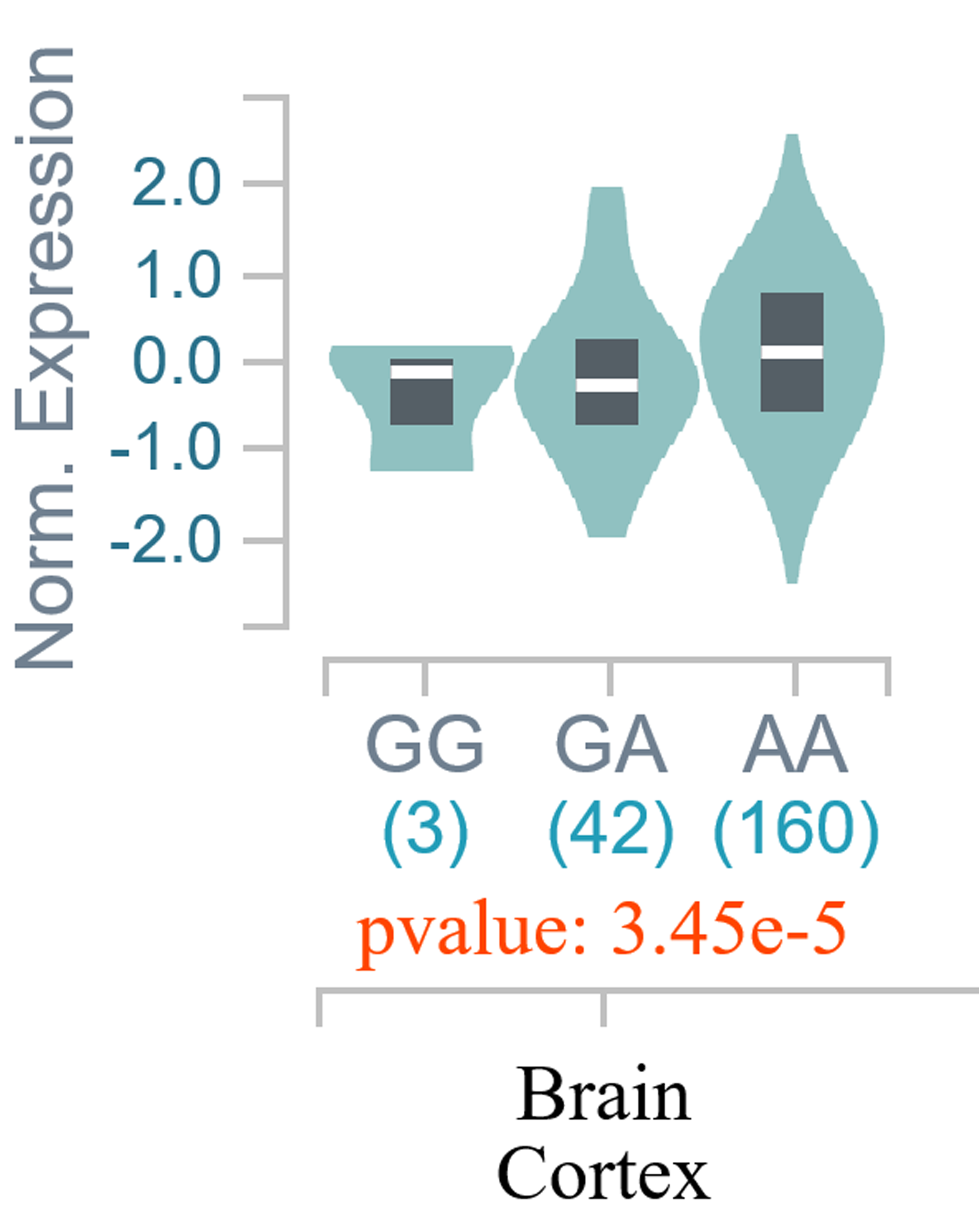


**Figure S1. Functional implication of** **rs8400 polymorphism based on the public database GTEx Portal.** The genotype of rs8400 and expression of *ALKBH5* gene in brain cortex.
